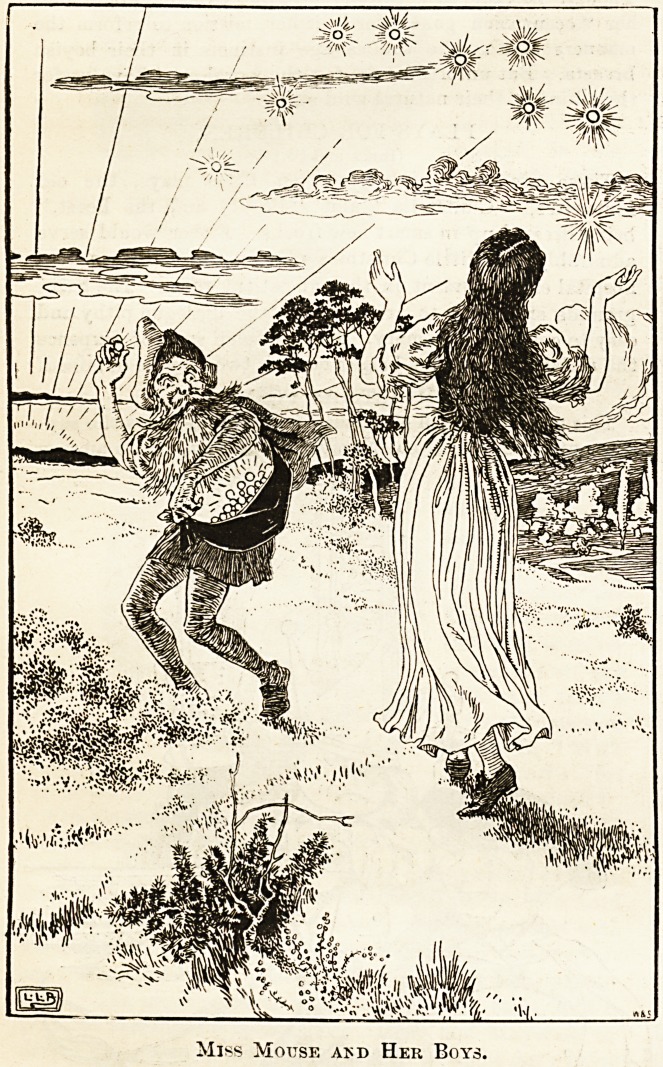# "The Hospital" Nursing Mirror

**Published:** 1897-12-18

**Authors:** 


### "The Hospital" Nursing Mirror.

Being the Nursing Section of "The Hospital."

[Contributions for this Section of "The Hospital" should be addressed to the Editor, The Hospital, 28 & 29, Southampton Street, Strand, London, W.C., and should have the word "Nursing" plainly written in left-hand top corner of the envelope.]

### News from the Nursing World.

### A CHRISTMAS GREETING.

We heartily wish our readers a very happy Christmas. It has lately been our frequent good fortune to receive many testimonies as to the value of The Hospital, and, surest sign of popularity, its circulation is rapidly increasing. We hear from matrons, burdened with the responsibilities of office; from probationers, just on the threshold of their work; and from all kinds of nurses in between. News comes from fur-clad toilers in the snowy north; from muslin-robed nurses in the sweltering south, and one and all bear witness to the help they find in our columns. Difficulties crop up in every path; and, even as every heart knoweth its own bitterness, so has each its own peculiar joy. Sympathy and co-operation, however, disperse many difficulties; they are said to halve sorrows and double joys; and it is our wish and aim to give to the world of nurses a centre of union and communion in the pages of The Hospital, and our readers can give us no greater pleasure than to make ample use of the opportunities thus offered.

### H.R.H. PRINCESS CHRISTIAN'S TRAINED NURSES.

The Master of St. Katherine's, president of Queen Victoria's Jubilee Institute for Nurses, gives the following report on the tenth year's work of H.R.H. Princess Christian's Trained Nurses: "We have received a most satisfactory report of the home and district work at Windsor and Eton. H.R.H., the president, and committee are to be congratulated on their beautiful home and excellent and efficient nurses." The staff consists of a lady superintendent, four district, and thirteen private nurses. There are branches at Chertsey, Eton, Addlestone, and Egham. The public have supported the homes liberally, and the demand for nurses has been in excess of the supply. Miss Simpson, who has been an able and popular superintendent for the past five and a-half years, has recently tendered her resignation, much to the regret of the committee.

### THE COLONIAL NURSING ASSOCIATION.

At the end of eighteen months' work the first annual meeting of the Colonial Nursing Association was held at the Imperial Institute. One of the most interesting features of the meeting was the account of the successful result of the pioneer nurses at the Mauritius and at Cyprus, both financially and from a nursing point of view. At the Gold Coast, too, the death-rate is considerably reduced by the services of the trained nurse. Lord Loch, the chairman of the meeting, asked the public to support the scheme generously, the total amount received so far being £709.

### A GOOD IDEA.

Miss Cash, matron of the Chelmsford Infirmary, has most kindly given an evening every week for the last two months to instructing the elder members of the Girls' Friendly Society in home nursing. About thirty associates and members were present at each lecture.

### OUR DISTRIBUTION FUND.

We are looking forward with great interest to our Christmas distribution of garments and useful things to the hospitals. Already many nice-looking parcels are on the office shelves waiting to be examined. The Editors of The Hospital have promised a contribution of £1 1s. Of course, piles of capital garments are given away at Christmas from many sources, but there are hundreds of poor folk who never get one, but we are sure that work we give to be distributed by the hospitals does reach the really destitute. Such gifts do double good—they help the poor, and likewise cheer the nurses, whose hearts ache badly when they see the patient whom they have coaxed back to health go out into the inclement streets scantily clad. All contributions please before the 22nd inst.

### GUY'S NURSES.

The first of February, 1898, will bring to the nurses at Guy's Hospital a considerable increase of liberty. The new regulations have been under discussion for some time with the result that the average working day, exclusive of Sunday, will be only 8½ hours. The arrangements of the new hours have been most thoughtfully considered. They are such as to enable the nurses to one day write their letters and darn their stockings, and the next to get quite a long outing. The sisters, too, get a couple of Sundays off in the month, the actual time being from four p.m. on Saturdays to ten a.m. on Monday mornings. A new class of probationers, who have already served one year, has begun work at pharmacy and dispensing under Mr. Collier. Such classes have always been held for the instruction of lady pupils, but it is a new departure to provide them for the regular probationers. This is quite in keeping with the decision of the authorities to promote nurses by seniority and not by social status as hitherto. Lady pupils who pass a successful novitiate have the same opportunity as other probationers, but are not favoured beyond them.

### THE NURSES' NEW HOME, GUILDFORD.

Nearly £3,000 was raised by the special appeal for the Royal Surrey County Hospital, Guildford. The Board of Management have therefore been able to reconstruct the system of drainage and to build the new nurses' home. The home contains a sitting-room, writing-room, and sixteen bedrooms, together with lavatories and bath-rooms. The dining-room and kitchens are retained in the old blocks. The rooms are connected by corridors running the full length of the building, the decorations are uniform, all the walls being painted a light blue; the bedrooms are comfortably furnished, and hot and cold water is laid on everywhere. The chief defect is that there is no covered way between the new and old buildings. As the estimated cost is £400 and there is no money this improvement must wait awhile. We hope not very long, as passing to and fro in damp weather cannot fail to be injurious to the nurses. The new home is named the "Henry Taylor," in memory of a medical man who was on the visiting staff of the hospital for many years, and who did much for the institution.

### New Year in a New York Hospital.

FROM A CORRESPONDENT.

THE observance of Christmas in the United States is more or less a new-fangled concession to English custom; and there is something of an "imported air" about the manner in which it is kept. Many of our customs, and our English Christmas cards especially, with their suggestions of red-berried holly and scarlet-breasted robins, are sources of curiosity to our Transatlantic cousins. For American holly bears no berries, and the Yankee robin, which is not unlike a small English partridge, has so innate a dislike of the cold weather we invariably associate with him that, at the first breath of wintry wind, he migrates post-haste to sunny and southern climes. It is, indeed, doubtful if he has ever seen snow. But there is no doubt that he would strenuously object to it as a background!

The honouring of New Year is, however, a real custom of the country, and it is done in national fashion. A few jottings from the memories of a New Year spent in a New York Hospital may prove of interest to English nurses who have not had the pleasure of a trip to the "other side."

From time immemorial it has been the custom in the United States for ladies with any social pretensions to be "At Home" to their masculine friends on New Year's Day, somewhat after the French fashion. But in America receptions used to begin as early as ten a.m., and lasted till midnight. After breakfast blinds were drawn, the gas lighted, and the ladies received their guests in full ball costume. The custom now is to make New Year calls on intimate friends only. But so recently as five years ago I can recall the almost total absence of femininity from the streets of New York on January 1st.

Gentlemen callers were expected to don swallow-tails and to offer New Year congratulations to all the ladies of their circle, and their congratulations were more welcome when accentuated by bouquets of lovely flowers and gigantic boxes of "candies." Champagne, ices, and other refreshments were served from early morning till midnight, and many a young man has confided to me the quantities of champagne he has consumed between the breakfast and luncheon of a New Year Day!

In our Hospital we were "on view" from a very early hour to friends and others who chose to call. And we received a goodly share of "candies" and flowers. Our freshest uniforms and prettiest caps were donned for the occasion, so it was not so necessary for us to shut out daylight as for the wearers of "low-neck and short sleeves."

A bright service in the chapel opened the day's festivities—on Christmas Day we had no service—presents were exchanged, and good-will and cheerfulness prevailed. Friends of the patients were admitted without restriction or limitation of any kind. A turkey dinner, with the usual "fixings" of celery and cranbery sauce, and the ice-cream and cake, without which no American function is complete, gave supreme satisfaction all round.

One custom which might be commended to the notice of committees in England was the presentation of a New Year cheque to all superior officials who had been over twelve months in the service of the hospital. The matron received the nice little sum of £10, while £8 fell to the lot of the assistant matron, and so on down a descending scale from dispenser to engineer. The gifts were acceptable to the nursing staff as contributing towards the many expenses for decorations and for the entertainment of the patients, which all festivals in hospital bring in their train. And there was no encroachment on hospital funds, for the sum necessary had been kindly subscribed by the lady managers themselves, and was offered in a pleasant spirit of appreciation of our work.

Great excitement had reigned throughout the hospital on New Year's Eve at the discovery on our doorstep of a three-week-old foundling. About half-past ten p.m. the senior house physician, on his way to the letter-box, found the little thing asleep just outside our front door. The night was keenly cold, and snowy, and the child was chilled despite its excellent clothing. The doctor looked round quickly for some trace of an owner, but the street was apparently deserted. We took the little thing in, and we had quite an imposing christening ceremony in the chapel on New Year Day. The house-physician stood godfather to his "treasure-trove," which he named after himself, while I was godmother and promised all sorts of good offices which were never to be fulfilled, for "our baby," who was quite the centre of attraction during the New Year festivities, died some three weeks later. The incident reads suspiciously like the stock stories of "Christmas numbers," but it is absolutely true. The entry of the circumstance, the naming and the death of the child is entered in my own hand on the register of the New York Children's Hospital at which it happened. It was such a fragile, fair little baby that we hardly thought when we took him in that he would live so long as he did.

We often speculated as to whether his mother ever enquired as to his fate, and some surprise was expressed that she should have brought him to us, for the New York Foundling was so near, and there he would have been received without question or comment. I learned that many a baby had been found on the doorstep of our hospital, but few had ever lived.

It was not easy to decide between the many nurse applicants for "leave" on New Year's Day. But it was all settled satisfactorily, leaving the wards as nurseless as was compatible with the patients' comfort. And those fortunate enough to be "off-duty" on so festive an occasion as New Year's Day, went off gaily sleighing in Central Park, and winding up the evening with theatre-party or dance.

My holiday had been taken—English fashion—at Christmas, so that I was pleased to take a turn in the wards while the nurses disported themselves in merry-making. I fear some good folk in England will be shocked to hear that I had yielded to the spirit of doing "as Rome does," and had joined a theatre party on Christmas night. It is the custom so to do in the United States, and there were thousands of good Americans that night in the New York theatres. But I, with my English training and tradition, had by no means "a good time." It will refresh the good folk to hear that I sat in that theatre so humiliated in conscience that I realised in person that description of Mark Twain of "feeling as if I had robbed a blind beggar in church."

But though my Christmas was a failure, I thoroughly enjoyed my new year in a New York hospital, and hope it will not be the last I shall spend in that pleasant country.

### Minor Appointments.

BOLTON INFIRMARY AND DISPENSARY.—Miss A. Baker, who was trained at Leeds General Infirmary and Monsall Fever Hospital, Manchester, was elected Sister of the children's ward on December 9th. Miss Baker has been charge nurse at the Brook Fever Hospital, Shooters' Hill, and for the last six months she has been engaged in private nursing.

CARDIFF UNION INFIRMARY.—Miss Frances Beardmore was selected Charge Nurse of the above institution on December 11th. She was trained at the General Infirmary, Hereford, where she was afterwards charge nurse. She was subsequently engaged in private nursing at Cardiff, and then became nurse at Martley Union.

### Christmas Fare for Invalids.

THIS is often a difficulty for nurses, not wishing their convalescing patients to be left out in the cold when all the rest are enjoying this festive season; and yet what can they have in place of the proverbial roast beef and plum pudding? Select any of the following menus and the patient will feel he or she has been treated to the most recherché of meals:—

MENU No. 1.

Whitebait. Chicken soufflés.
Sponge pudding. Cheese wafers.

MENU No. 2.

Oysters. Brown bread and butter.
Fillets of partridge. Chip Artichokes.
Orange compôte. Anchovy biscuits.

MENU No. 3.

Creamed fish. Roast pheasant.
Meringues. Ramequins.

RECIPES.

WHITEBAIT.—These little fish must be perfectly fresh and kept with a little ice on them. Drain on a sieve, but do not wash them. Take about ten or fifteen and place them on a clean cloth and sprinkle with flour. Shake them so that all are evenly covered with a very thin coating of it. Put them in a frying basket, and fry in clean, boiling fat for one and a half minutes. Just before serving plunge them in again into the boiling fat, and fry for two minutes. Serve very hot on a dish paper and hot dish, with brown bread and butter and a quarter of a lemon.

CHICKEN SOUFFLÉS.—The breast or leg of a raw chicken are best for these. Scrape off all the meat, chop and pound it well, and rub it through a wire sieve. To one ounce meat allow the yolk of one raw egg, a little pepper and salt, mix well together in a basin, then add a table-spoonful of whipped cream and the white of an egg stiffly whipped with a pinch of salt. Butter two small soufflé cases or more, three parts fill them with the mixture, and bake in a moderate oven for twelve minutes.

SPONGE PUDDING.—Well butter a half-pint mould and garnish the sides and bottom with glacé cherries and angelica cut into small strips, then line the mould with slices of stale sponge cake, and fill up with a custard made with two whole eggs mixed with a quarter of a pint of milk and one ounce of castor sugar. Put a buttered paper over the pudding, and tie down tightly with a cloth. Steam it for three-quarters of an hour.

CHEESE WAFERS.—Rub a quarter of an ounce of butter into two ounces of flour, add half an ounce of grated Parmesan cheese and a little coralline pepper, mix into a stiff paste with a little cold water, and roll out as thin as a sixpence, prick the paste with a biscuit pricker, and stamp into rounds with a round cutter. Place the rounds of paste on a baking tin and bake in a moderate oven for five minutes, when they should be quite crisp and a light brown colour.

ORANGE COMPÔTE.—Line a fancy mould with lemon jelly and arrange neatly some quarters of oranges, quite free of pith and pips, and set these with a little liquid jelly, then add more, pouring in the jelly when the last lot is quite set. When wanted for use dip the mould in warm water, wipe the top with a clean cloth, and turn out the jelly into a glass dish.

CREAMED FISH.—Take half a pound of fresh turbot, and place it in a baking tin that has been slightly buttered; season the fish with a little salt and a few drops of lemon juice. Pour over it a quarter of a pint of single cream, and cover it with a buttered paper. Place the tin in another containing boiling water, and bake in a moderate oven for twelve minutes, basting it constantly. Dish up on a hot dish, pour the cream liquor over it, and sprinkle with chopped parsley.

RAMEQUINS.—Mix in a basin one yolk of an egg (raw), a tablespoonful of milk, a teaspoonful of grated Parmesan cheese, a little pepper and salt, and the white of the egg stiffly whipped. Three-parts fill some ramequin cases, and bake in a moderate oven for about six minutes.

### Nursing in China.

### GOVERNMENT GENERAL HOSPITAL, HONG KONG.

ENGLISH nurses are well acquainted with the type of patient who, having spent one Christmas in hospital, makes himself conspicuous on arrival by informing his companions of the pleasures he enjoyed on his former visit. "When I was here before," prefaces many flowery statements, and especially those he makes with regard to the great Christmas festival. This old patient is often the unconscious medium through which new comers learn that they are uncommonly lucky to have come into hospital in December, an idea which probably had not previously presented itself.

But the return of former patients is not a circumstance peculiar to English hospitals, and one of these old patients happened to find himself settled in the European wards at the General Hospital at Hong Kong at the end of 1895, and he forthwith proceeded to favour the new occupants of the beds with sundry reminiscences of his own.

Foremost amongst the pleasant memories on which he expatiated were the carols sung by the nurses. He told how these English ladies had started at six in the morning, and gone round the wards and verandahs delighting their countrymen, who were, like himself, so far from their native land.

To the disgust of this old patient he learnt that it was not proposed to repeat the early carol singing this year, but those whose hopes he had raised by his description altogether declined to be baulked of the entertainment. They quietly asked first one official and then another, until on Christmas Eve they obtained the coveted permission. The nurses added a preliminary practice to their other labours, and on Christmas morning the patients were gratified by carols, which probably sounded all the sweeter for the old memories by which each familiar tune was surrounded.

The next event was the appearance of a little figure in a peaked cap, round which was a band bearing the letters "Postman." But this was no ordinary letter carrier, and the contents of his bag consisted entirely of Christmas cards, provided for the patients by the nurses. As for the postman himself, he was no common conventional official. He was just a wee creature who, having spent the greater part of his short life in hospital, was now hobbling about on crutches. It was a kind thought to give to such a child on Christmas morning the pleasure of being useful, and to confer on him the temporary importance of personating "our own postman."

When the small cripple had completed his delivery of letters he did a little bit of private business on his own account, and when his nurse went to her room she found tied on to the handle of the door a small stocking, containing some sweets, oranges, and an apple, which showed that the little postman did not wish his best friend to be left out in the cold. The tenants of the sailors' ward had their Christmas dinner at a prettily set table in the verandah, from which a charming view of the harbour is obtained. They had excellent mulligatawny soup, fish, turkey and sausages, plum pudding, and mince pies.

Meanwhile the sailors' ward had been converted into a concert-room, where a musical entertainment took place from half-past three to half-past five, which was thoroughly appreciated. The dinner in the evening for the staff was a most successful gathering, and the table was lovely with a wealth of flowers, and the only speech of the evening was, "Friends at Home and Sister Gertrude," the latter being the name of a valued nurse to whom sick leave had been granted.

The hospital itself is picturesque, and it is beautifully kept; the trained nurses, whom Miss Eastman superintends with so much ability, have native assistants to do the work of orderlies and ward maids.

### Nurses at Maidstone: The Mayor's Reception.

THERE are still many nurses in Maidstone, and the new cases of typhoid notified from day to day are likely to make the presence of a certain number necessary for some time to come.

The emergency hospitals continue open, although one or two may shortly be dispensed with, and there is no chance of the nursing being over for at least several weeks more.

The number of nurses has, however, become perceptibly reduced, especially of those engaged in district work. Before the majority left the town the inhabitants desired to give to each a lasting souvenir of the place. A subscription was started privately amongst the townspeople, who were unanimous in their support of the plan, and a design for a silver medal was chosen. On one side the handsome arms of the town were reproduced; on the other the nurse's name, the date, and the words, " With gratitude for loving services."

The Mayor and Mayoress of Maidstone issued invitations for a reception to be held on the 8th inst. in the museum, a particularly handsome and interesting old mansion with an *annexe* containing excellent technical schools. The Lord Mayor of London, himself a Kentish man, was present with his daughters and the Sheriffs, also several provincial mayors and a large number of guests from the town and neighbourhood. The arrangements were in excellent taste, and a pleasant evening was spent. The Mayor and Mayoress received the guests on arrival, the nurses on night duty being amongst the earliest of these. They were marshalled to their numbered seats in the hall, where the presentation of medals was made by the Mayor. An interesting address by the Lord Mayor followed, and Mr. Arthur Urmston returned thanks for the nurses. Owing to the number of speeches which followed the actual ceremony, the night nurses had to leave immediately on their conclusion, without seeing anything of the charming museum. The day nurses, whom they went away to relieve, fared better, as they were able to stay until the conclusion of the evening, and heard some good music and saw an exhibition of the ever-popular "animated photographs."

The medals were bestowed on the nurses employed by the Corporation; on Miss Jones, matron of the West Kent Hospital, and the ward sisters, who had all been engaged with typhoid cases, including the sick nurses. Miss Travis, permanent district nurse at this hospital, also received a medal in recognition of her services in superintending the emergency nurses' district work. Miss Plowman, matron of the temporary hospitals, and the night superintendent of the "Relief" nurses, also received medals. The Lord Mayor, Sir John Monckton, and other officials were also presented with this token of the town's gratitude, and one was handed to the curator to be preserved "in the archives" of the museum. About 250 medals were struck and no other copies will be procurable. Many nurses who have worked through the epidemic were ineligible as recipients by reason of their being employed by individuals and not by the Corporation. In some cases kindly benefactors supported nurses for special poor parishes. It was said that the employers of private nurses could purchase medals (if they wished to do so) for those who had served them well.

The air of friendliness and good-will which pervaded the Mayor's unique reception was well defined. Nurses were heard eagerly introducing their friends to "the lady who has had two nurses as visitors all the time, and hasn't she just spoilt us with kindness." Town Councillors pointed out the special nurses with whom the illness of near relatives had brought them into contact, and so on. Resident as well as emergency doctors greeted nurses, to whom they frankly owned their gratitude for faithful service, rendered in emergencies when medical and nursing skill combined, had rescued "bad cases" from the very gates of death.

As for failures in the nurses themselves, they have been so rare as to make them remarkable, and the bereaved families have continued the true nurses' most grateful friends and have taken an unselfish interest in sanctioning (although they could not join in) this public recognition of their usefulness. The way in which the majority of the trained nurses worked at Maidstone, especially in the first weeks, when the demands on skill and strength were almost incessant, will not easily be forgotten by those who witnessed the daily unselfish sacrifice of personal inclination and comfort.

### In a Christmas Library.

### THE CHILDREN'S SHELF.

"We get more books at Christmas than we used to," said a wise little maiden of eight years, "and not nearly so many mince-pies and goodies." The latter part of the sentence

The Wallypug in London.

was uttered in most rueful tones, as of an octogenarian recalling the delights of youth. But it is a truth that each season tends to translate the good cheer of Christmas to more intellectual and artistic planes. And the plum puddings and port of the past transmute themselves into the lovely gift-books of to-day. With so much that is delightful to choose from in Christmas books it is a little depressing to remember that this is not the season for additions to hospital or nursing libraries, unless some good publishers will take it into their heads to act the part of fairy godmother or generous Santa Claus and send a timely volume or two to nursing libraries. For Christmas entertainments, flowers, and sumptuous ward teas absorb the spare—and other—cash in nursing pockets. No surplus remains for books—charm these ever so delightfully—save those which are destined as presents for others.

### THE WALLYPUG.

(Methuen and Co.)

On the children's shelf precedence must be given to "The Wallypug in London," for it brings out an incident of a clever little maiden of nine years old who thus early determined she would not remain on the shelf. The author, Mr. Farrow, tells in the preface how this enterprising little damsel wrote asking him to "wait for her till she grows up, as she would like to marry a gentleman who told stories." She is evidently a new woman in embryo; but after reading "The Wallypug" it is easy to forgive her effort to secure for life so charming a fireside story-teller. May she choose with equal discretion in her maturer years. The many boys and girls who delighted in "The Wallypug of Why" will be delighted with their old friends' Jubilee visit to London and their journeyings to the Tower and Madame Tussaud's. The illustrations are excellent, as may be seen from the specimens given—not quite equal, however, to those of Mr. Furniss and daughter in the previous volume—and the brightness and wit of the letterpress never flags for a moment.

### MISS MOUSE AND HER BOYS.

(Macmillan and Co.)

Miss Mouse is a delightful little girl of Mrs. Molesworth's creation, and everybody familiar with her books, which should be on the shelves of every well-conducted nursery, knows she can depict most charming small persons of either sex.

Little Miss Mouse has a retinue of admiring boy cousins who do their best to convert her into a spoilt beauty. "Her boys" are a wild, unruly set. By the bye, it is rather a pity that most books written for children represent boys as so beset with the original sins of roughness and rudeness that life with them is well-nigh unbearable. As Miss Mouse says after a tea-party with her boys, "My head feels rather 'buzzy,' I think."

Ours felt "buzzy" after simply reading about them. Surely boys might be boys after a gentlemanly fashion. Though in that case Miss Mouse, like Othello, would find her "occupation gone," for it is her mission to reform the manners and soothe the savage instincts in their boyish breasts. But unfortunately for the moral most boys prefer their kind in their natural wild state.

### PLAYS FOR CHILDREN.

(Innes and Co.)

These are two brightly-written little plays, the old favourites, "Cinderella" and "Beauty and the Beast," being dressed up in smart new frocks. Either would serve admirably for a little Christmas play in the wards or at a hospital entertainment in the out-patient room. The backgrounds are simple to arrange, and the dialogue pithy and easy to commit to memory. For the small sum of sixpence the material for a very bright hour or two may be obtained.

### CHRISTMAS POSY.

This pretty "bunch of verses" for children forms the supplement to the Christmas Number of *Woman*. With an easy

The Wallypug in London.

rhyming jingle and attractive pictures it is an admirable sixpennyworth. Looking at its many charms it is pleasant to know that the proprietors of *Woman* have generously distributed a special edition of this fascinating "bunch o verses" as a Christmas gift to the principal homes and hospitals for children.

### A BOOK OF NURSERY RHYMES.

(Methuen and Co.)

Here we find the old favourites of our childhood dressed up anew and attractively. Goosie, Goosie, Gander disports himself in the most impossible of gay artistic gardens, wherein little boys in smocks form decorative cavaliers attendant on wee maidens in peacock blue with golden-hair halos like little saints in stained-glass windows. Little Boy Blue has his attendant cows in the meadow and sheep in the corn, illustrated with that delightful time-honoured perspective which makes the objects the farthest

Miss Mouse and Her Boys.

off appear the largest in the picture. Any child who would not enjoy this book deserves to be smacked for *blasé* grown-upness.

### FOR THE QUEEN'S SAKE.

(Thomas Nelson and Son.)

This is a specially interesting children's book, as the name of E. Everett Green on the title-page leads one to expect. describes the coming of a charming little orphan boy from India to his old grandmother, occupying one of the stately homes of England. The troubles and joys of his life, and the companionship of his dog, are very well given.

### A CLERK OF OXFORD.

(Thomas Nelson and Son.)

Older children will be delighted with "A Clerk of Oxford," by the same author—a book with an historical motive, since the clerk meets with exciting adventures in the War of the Barons. The pen pictures of Old Oxford are specially charming, and though many young people take exception to a story-book with an undercurrent of teaching—though this be wrapped up pill fashion in the sweetest possible coating—few would exercise the spirit of modern criticism so strongly as to look such a gift-volume in the mouth. We recommend it as a Christmas present to good or bad alike, for often one has a very soft corner in one's heart for those whom a small boy once described as "the scapegoats of families." "A Clerk of Oxford" will tempt many young readers to keep late holiday hours. The Battle of Lewes and Christmas at Kenilworth are specially well done. The charm for grown-ups is indicated by the fact of the reviewer being tempted to linger much longer over the book than is discreet when the Christmas bookshelf is crowded with volumes calling for criticism.

### VANDRAD THE VIKING.

(Thomas Nelson and Son.)

Breathes there a boy with soul so dead to glory that all the martial spirit in him is not roused by a recital of the wondrous deeds of the Vikings. This book of stirring fights by land and sea and wondrous victory will stir the hearts of all those boys to whom Christmas morning brings so congenial a gift as the present volume.

A general survey of children's books suggests that the art of writing for the young is not keeping pace with the standard of other books. In America literature for children is of a delightful order. There the small folk have their newspapers, their magazines, and an array of charming writers all to themselves. Here one can count successful writers of children's books on the fingers of one's hands. Which reminds us of the sad fact that Mrs. Hodgson Burnett—who has given us two delightful children's books—has quite forsaken the little folk, which is hard both on the little folk and the big.

### A SHELF FOR THE GROWN-UPS.

### THE QUIVER.

The bound volume of the *Quiver* for 1897 is of unusual excellence, and would be a welcome addition to a hospital library. It contains many articles of interest—not invariably written in the tone of instruction, the "I give you this information for your good," which acts as a ready stop to the brain cells. There is an interesting article by an English nurse of her Christmas spent in nursing a little Arab through typhoid fever—her valiant struggles to give the patient the necessary washings being amusingly described. One cannot but pity the trials of a distracted nurse who must needs gain permission from eight feminines, speaking a language she understood not, before carrying out the simplest routine of the sick-room. After a persistent struggle, and the enlistment of the father's services, she succeeded triumphantly in getting her patient into a nightgown. For many days and nights he had been fully dressed in frock and petticoats. She was not altogether sorry when his convalescence resulted in her departure from this strange Coptic household.

### CASSELL'S FAMILY MAGAZINE.

(Cassell and Co.)

This is not quite so interesting as the *Quiver*, but it contains attractive stories by Bret Harte, Hon. Mrs. Henniker Heaton, Robert Barr, L. T. Meade, and other writers of note. The illustrations throughout are good, and the letterpress up to the well-known good standard of all Messrs. Cassell's publications.

### THE WHITE SHIELD.

(Cassell and Co.)

This is a reprint, the book having been published first in 1895, and is one of the martial Zulu stories in which Bertram Mitford delights. The narrative is of a great Zulu chief, and it is unfortunately told in that semi-allegorical language which soon makes very tiresome reading. This fashion for allegory is too cumbersome to suit practical, busy nineteenth century workers, who prefer the plain everyday speech of humanity.

### THE SINGER OF MARLY.

(Cassell and Co.)

May be highly recommended as a bright, adventurous romance. The hero and heroine are most delightful Irish people, and the result of the sojourn of Bridget, a most loveable product of the Emerald Isle, at the Court of Versailles, in company with Madame de Maintenon, is extremely well told. The beautiful Irish girl wins all Parisan hearts, but hers beats true to her Irish lover. The love story is idyllic and delightful. A hypnotist and the enchanted spells he casts over the "sensitives" coming under his influence will prove specially interesting to some readers.

### THE HIGHWAY OF SORROW.

(Methuen and Co.)

This is an extremely interesting story, setting forth the little known history of the Stundists, the religious peasants of Russia, whose Christianity is as simple as was that of the early Apostles. Miss Stretton has been assisted in her work by a Russian exile, to whom she is indebted for her graphic scenes of Siberian prison life. To all who care for the religious novel this book may be commended with confidence.

### THE OUTLAWS OF THE MARCHES.

(Cassell and Co.)

Of the making of Scotch books there seems no end, but so long as these are so good as the above their manufacture may go on indefinitely. A volume such as this will add a fresh charm to the Christmas holidays, and "gin a body" were to ask the reviewer to name one of the six books desirable for Christmas presents, "The Outlaws of the Marches" would be one of them.

### "LOCHINVAR."

(Methuen and Co.)

Crockett's "Lochinvar" would also be "put on the list" as a charming combination of Scotch and Dutch. It is a new departure for Crockett to go to the Continent for his history and adventure. Surely the romance stored up in the bog and peat land is not coming to an end? However that be, no lovers of Crockett will regret this delightful story of the Prince of Orange nor the scenes of Holland with which some of the setting is arranged, for there is enough of Scotland and Highland outlawry to satisfy the most ardent followers of Scottish romance and chivalry. "Lochinvar" should be writ large on the library lists of books wherewith to make Christmas a happy season of interesting reading.

### Help the Nurses to Help the Sick.

**Royal National Pension Fund for Nurses,** 28, Finsbury Pavement, E.C.—This Fund has during the past year maintained the uninterrupted success which has attended it ever since its establishment. The number of new members who have joined the Pension Fund in 1897 has been considerably above the yearly average. Over £1,200 has been distributed in sick pay, a fact which speaks eloquently as to the blessing which this branch of the Fund's work must be to nurses, more especially, of course, to those working on their own account. More than £1,700 was paid away in pensions, against £1,279 in 1896, £817 in 1895, and £607 in 1894, showing how greatly the Fund is increasing its sphere of usefulness. The premium income, *i.e.*, the payment by or for nurses, exceeded the enormous total of £56,000.

**The Junius S. Morgan Benevolent Fund** is an auxiliary to the above fund for nurses, and was founded through generous contributions from nurses themselves, and raised to handsome proportions by the munificence of the Morgan family and many other friends to nurses. The work is done by volunteers, under the supervision of an influential committee, which devotes time and care to the investigation of claims and the relief of urgent cases. Hon. Secretary, Miss Rosalind Pritchard.

**East London Nursing Society.**—The object of this society is to nurse the sick poor in East London in their own homes by means of trained resident nurses, each nurse living in the parish in which she works. The extent of the society's useful work is shown by the fact that in 1896 the staff of 33 nurses attended to 4,984 persons, to whom 121,689 visits were made. Annual subscriptions and donations to the general fund are asked for. Secretary, Mr. Arthur W. Lacey, 49, Philpot Street, Commercial Road, E.

**Metropolitan Nursing Association,** Bloomsbury Square, W.C.—Founded in 1875 as a Training School and Home for District Nurses who have previously gone through a full course of hospital training, and who nurse the sick poor in their own homes within a radius of a mile and a half from Bloomsbury Square. This Association is now the central training home for the Queen's Nurses. Superintendent, Miss Gray.

**Queen Victoria's Jubilee Institute for Nurses.** Offices at St. Katharine's Hospital, Gloucester Gate, Regent's Park, N.W.—The institute trains nurses in district nursing, and supplies nurses for the sick poor in their own homes. Applications for information should be addressed to Miss Peter, the Inspector. Nursing associations in Scotland, Ireland, and Wales are affiliated with the institute.

**Up-Country Nursing Association for Europeans in India**.—The chief object of this association is the provision of skilled nursing for Europeans, especially civilians, in the up-country districts of India. The association in London selects the nurses, and pays all their expenses until they arrive at their destination in India. Once established there, the cost of maintaining the nurses falls upon the Local Committee. Hon. Secretary, Major-General J. Bonus, R.E., The Cedars, Strawberry Hill.

**"The Hospital" Convalescent Fund.**—Since the establishment of this fund many tired and delicate workers have enjoyed a much-needed change of air such as they could not possibly have secured for themselves without help. Experience has proved that it is better to let the nurses have a choice of locality rather than to send them to one settled place, and the object of the fund, namely, to provide rest for weary workers, amidst suitable surroundings, without any of that anxiety about ways and means which retards convalescence is fully carried out. Contributions which would increase the field of usefulness are invited by the Hon. Secretaries, care of the Editor of THE HOSPITAL.

### Where to Go.

BENEVOLENT FUND, ROYAL BRITISH NURSES' ASSOCIATION.—Miss Maude Danks will give a grand evening concert at St. James's Hall on December 20th. Tickets may be had from the Secretary, R.B.N.A., 17, Old Cavendish Street, W.; and from the Secretary, Victoria Commemoration Club, Southampton Street, Strand.

### Christmas Presents and Cards.

CHRISTMAS CARDS AND CALENDARS.

MESSRS. MARCUS WARD's publications this season are charming. They have provided something for everyone, and purchasers will do well to ask to see their cards, calendars, and almanacks before finally selecting. They have a great variety of the small calendars so convenient to carry in the purse or pocket book. They all bear the season's greetings, so form the nicest possible form of Christmas card. Some of the floral cards are very pretty, and an effective gold seal in the corner gives a very choice appearance. Messrs. Marcus Ward have been especially happy this year in their selection of words which express sentiments beyond the mere season's wishes. Aptly turned messages to absent friends, or lines recalling past times, are just needed at Christmas time, and here they are to hand. Very artistic are the little black and white landscapes, and the cards with religious designs in outline. Some greetings on parchment with illuminated lettering especially take our fancy, whilst in juvenile subjects the variety of designs is endless. But we must not forget the wealth of beautiful calendars before us. "The English Cathedral" calendar is formed of pictures each worthy of a frame to itself. The same may be said of the exquisitely got up "Romeo and Juliet" calendar, whilst the "Floral" is one of the prettiest of the kind we have ever seen. These are all very large hanging calendars, which form handsome Christmas presents, and very welcome ones too. There are very pretty ones much smaller, and also the very useful calendars with a fresh text, verse, or quotation for every day. One of these bears the portrait of the Princess of Wales, and this we predict will be popular amongst "Her Nurses." We could point out all sorts of other delightful Christmas specialities, but space does not permit.

Christmas shoppers this year will find no difficulty—except it be in an *embaras de richesses*—in something to suit every taste they desire to please, however small the means at command may be. The larger drapers offer real facilities by preparing bazaars, as they call them, where the wants not only of the youngsters are anticipated, but all sorts of pretty and useful knick-nacks are displayed to catch the eye of the elders, and spare them the trouble of going farther afield. Handkerchiefs, gloves, and Eau de Cologne are staple commodities, so to speak, in the way of presents, for they are always useful and always acceptable. Robinson and Cleaver, who now have a beautiful shop in Regent Street, have a special display of silk handkerchiefs for gentlemen, a hint that will be welcomed by many, as gifts for male friends are always a difficult problem. At 62, New Bond Street, is to be found that delightful Eau de Cologne known as "4,711," manufactured by Messrs. Mühlens, who are also celebrated for their Rhine Violets perfume. For our poor neighbours the cotton blankets that are so warm and so inexpensive will come in most appropriately; and the District Nurses' basket can be enriched at small cost by some packets of Chivers' Jelly, which will provide that exceedingly difficult article, namely a tempting and wholesome luxury for the invalid in poor circumstances which is not dependent upon careful or skilled cooking to render palatable. Every grocer keeps these jellies, so there is no difficulty in procuring them.

### Wants and Workers.

[THE attention of correspondents is directed to the fact that "Helps in Sickness and to Health" (Scientific Press, 28 & 29, Southampton Street, Strand, London, W.C.) will enable them promptly to find the most suitable accommodation for difficult or special cases.]

FLORENCE LAWSON, Toronto, Drummond Road, Boscombe, Bournemouth, will be glad to know of a second-hand edition of Quain's "Medical Dictionary." It must be of comparatively recent date.

### Everybody's Opinion.

[Correspondence on all subjects is invited, but we cannot in any way be responsible for the opinions expressed by our correspondents. No communication can be entertained if the name and address of the correspondent is not given, as a guarantee of good faith but not necessarily for publication, or unless one side of the paper only is written on.]

A MENTAL ATTENDANT'S OPINIONS.

MESSRS. MEAR AND FOWLER write: Our client, Mrs. Bedford Fenwick, has consulted us with reference to a letter appearing in THE HOSPITAL "Nursing Mirror" of the 11th inst., headed "A Mental Attendant's Opinion." That letter contains the following statement: "Mrs. Bedford Fenwick has said that it would be a disgrace for the R.B.N.A. to admit as members women who devoted their lives to the cause of the insane, otherwise, mental nurses." That statement is absolutely untrue, and as your correspondent cannot possess one iota of proof in support of it, it must have been made with the deliberate intention of misleading your readers. The animus of the writer is so evident that further comment appears to be unnecessary. Perhaps you will be good enough to publish this letter in your next issue.

### Appointments.

MATRONS.

ST. GILES' INFIRMARY, CAMBERWELL.—On December 8th Miss Bessie Hamilton was appointed Superintendent of Nurses at this infirmary. She was trained at Charing Cross Hospital, and has taken the L.O.S. diploma. Her experience is varied. She was a staff and private nurse at Charing Cross Hospital and St. John's House, and has been superintendent nurse at Stepney Union Infirmary and at Watford Union Infirmary.

DURHAM COUNTY HOSPITAL.—On December 7th Miss Dora Pressland was appointed Matron of Durham County Hospital. She was trained at the London Fever Hospital and Cardiff Infirmary, after which she took charge of Hatfield Broad Oak Hospital, and then she became assistant matron of the Royal Infirmary, Newcastle-on-Tyne, from 1890-5. Miss Pressland has excellent testimonials.

SWINDON ISOLATION HOSPITAL.—After completing her training at St. Bartholomew's Hospital, Miss Plumbly, the new Matron of the above hospital, became ward sister of St. Mary Abbotts Infirmary, Kensington, and subsequently night superintendent Monsall Fever Hospital, Manchester. She was elected for her new post on the 7th inst.

FORRES LEANCHOIL COTTAGE HOSPITAL, N.B.—Miss Agnes Reid, who was trained at the Royal Infirmary, Aberdeen, was appointed Matron of this hospital on December 4th, 1897.

### Notes and Queries.

*South Africa.*

(95) I want to go to South Africa as a district or hospital nurse. What is the best way to secure an appointment? Is writing direct to the homes and hospitals any use?—*Sister Bessie.*

Yes, you might write to hospitals and homes that you know are properly managed. You might also apply to the Colonial Nursing Association, Imperial Institute, W., and to reliable nursing associations in London, as they sometimes secure engagements abroad for nurses; or you might hear of a patient needing the services of a nurse who is going out, by one or another of these means or by advertisement.

*Address Wanted.*

(96) Two most daintily fashioned child's night dresses were sent some weeks ago to the Editor. Unfortunately, as the address of sender was not stitched on to them it has been mislaid. Will the owner therefore kindly claim her property?

*Invalid Cookery.*

(97) Can you recommend a good handbook on foods, and one that contains recipes for invalid cookery suitable for training nurses?—*Julia Sanders.*

Miss Maude Earle has just published a new book which is very good—"Sick Room Cookery and Hospital Diet, with Special Recipes for Convalescent and Diabetic Patients," price 3s. 6d. (Spottiswoode and Co.)

ANSWERS INVITED.

*A Cancer Case.*

(98) Will anyone give me any hints upon nursing a bad case of uterine cancer? The patient is always wet, and consequently suffers from bed sores. She has the use of a water pillow, but pads are too expensive for district work, and would be useless here.—*District Nurse.*

## Figures and Tables

**Figure f1:**
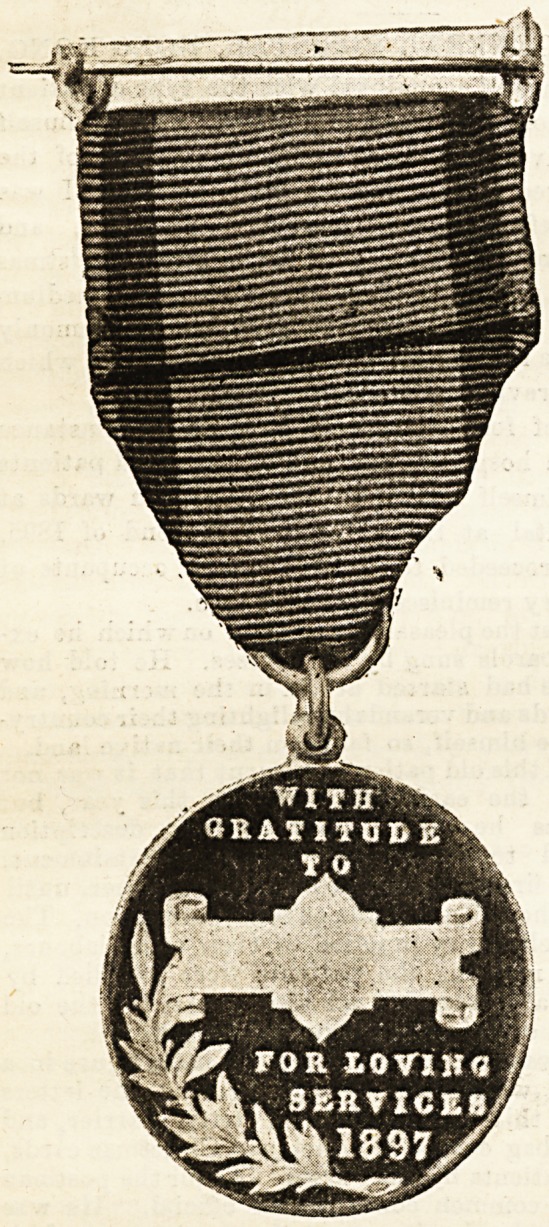


**Figure f2:**
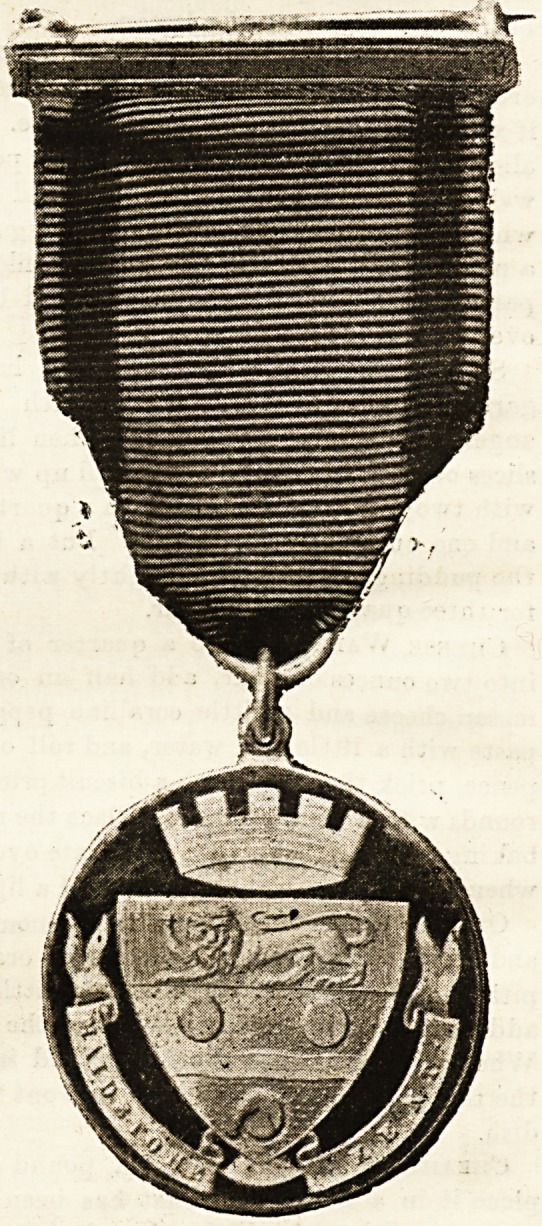


**Figure f3:**
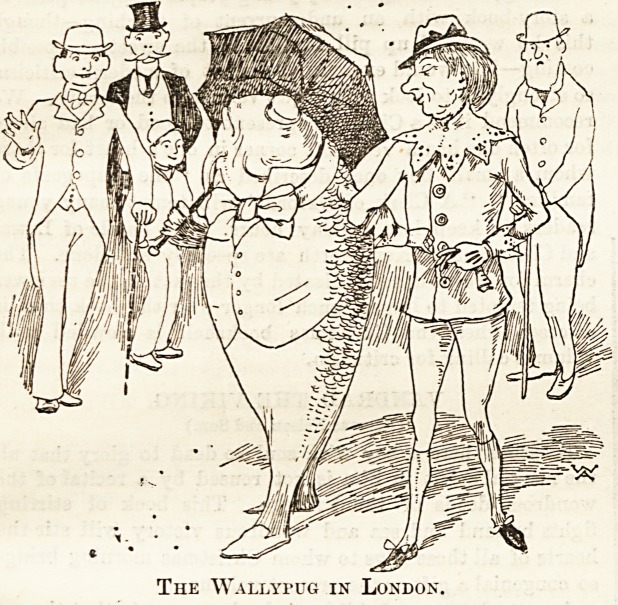


**Figure f4:**
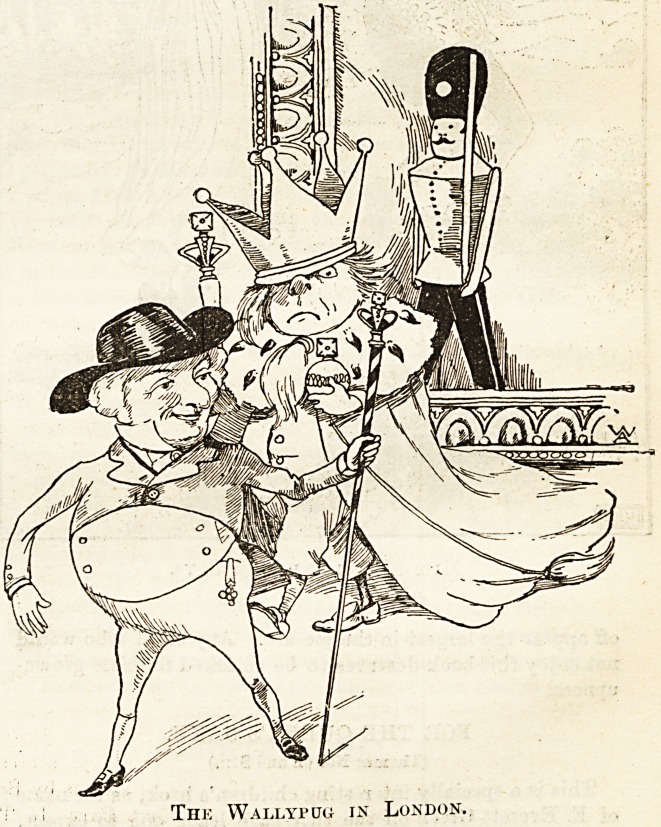


**Figure f5:**